# Sex-Specific Associations between Gut Microbiome and Non-Alcoholic Fatty Liver Disease among Urban Chinese Adults

**DOI:** 10.3390/microorganisms9102118

**Published:** 2021-10-08

**Authors:** Jiajun Shi, Yaohua Yang, Wanghong Xu, Hui Cai, Jie Wu, Jirong Long, Qiuyin Cai, Wei Zheng, Charles R. Flynn, Xiao-Ou Shu, Danxia Yu

**Affiliations:** 1 Department of Medicine, Division of Epidemiology, Vanderbilt University Medical Center, Suite 600, Nashville, TN 37232, USA; jiajun.shi@vumc.org (J.S.); yaohua.yang@vumc.org (Y.Y.); hui.cai@vumc.org (H.C.); jie.wu@vumc.org (J.W.); jirong.long@vumc.org (J.L.); qiuyin.cai@vumc.org (Q.C.); wei.zheng@vumc.org (W.Z.); xiao-ou.shu@vumc.org (X.-O.S.); 2 Department of Epidemiology, School of Public Health, Fudan University, Shanghai 200433, China; wanghong.xu@fudan.edu.cn; 3 Department of Surgery, Division of General Surgery, Vanderbilt University Medical Center, Suite 600, Nashville, TN 37232, USA; robb.flynn@vumc.org

**Keywords:** non-alcoholic fatty liver disease, gut microbiota, prospective cohort study, Asian population

## Abstract

Non-alcoholic fatty liver disease (NAFLD) has been linked to altered gut microbiome; however, evidence from large population-based studies is limited. We compared gut microbiome profiles of 188 male and 233 female NAFLD cases with 571 male and 567 female controls from two longitudinal studies of urban Chinese adults. History of NAFLD was assessed during surveys administered in 2004–2017. Microbiota were assessed using 16S rRNA sequencing of stool samples collected in 2015–2018. Associations of NAFLD with microbiome diversity and composition were evaluated by generalized linear or logistic regression models. Compared with controls, male cases had lower microbial α-diversity, higher abundance of genera *Dialister* and *Streptococcus* and *Bifidobacterium* species, lower abundance of genus *Phascolarctobacterium*, and lower prevalence of taxa including order *RF39* (all *p* < 0.05). In contrast, female cases had higher α-diversity, higher abundance of genus *Butyricimonas* and a family of order *Clostridiales*, lower abundance of *Dialister* and *Bifidobacterium* species, and higher prevalence of *RF39*. Significant NAFLD–sex interactions were found for α-diversity and above taxa (all false discovery rate < 0.1). In conclusion, we observed sex-specific gut microbiome features related to history of NAFLD. Further studies are needed to validate our findings and evaluate the health effects of NAFLD-related gut microbiota.

## 1. Introduction

Non-alcoholic fatty liver disease (NAFLD) is a highly prevalent metabolic disease, defined as ≥5% hepatic steatosis, not caused by excessive alcohol consumption or other secondary conditions such as viral hepatitis or hereditary liver diseases [1]. The estimated global prevalence of NAFLD was 25%, which varied significantly across countries from 4% to 41% [2]. In China, the prevalence of NAFLD has doubled in the past 20 years with a nationwide prevalence of 29% estimated in 2019; meanwhile, the prevalence was 33% in males and 22% in females [3]. The pathophysiology of NAFLD is complex; however, the gut–liver axis, i.e., the bidirectional relationship of the gut and its microbiota with the liver, has attracted increasing attention [4,5]. Gut microbiota can be involved in NAFLD development and progression through several mechanisms, including changing intestine permeability, changing energy harvest from diet, affecting lipogenesis and choline and bile acid metabolism, producing ethanol in the intestine, and linking to inflammation [4,6,7].

Recent animal models and human studies have linked gut dysbiosis with NAFLD [4,6]. Experiments using gut microbiota transplantation to germ-free mice showed that gut microbiota determine the development of NAFLD independent of obesity [8]. In addition, inflammasome-mediated gut dysbiosis was shown to be involved in NAFLD progression to non-alcoholic steatohepatitis (NASH) [9]. In human studies, as summarized by Safari and Gerard [4], several case-control studies have shown altered abundance of fiber-fermenting and inflammation-modulating bacteria, including *Dorea*, *Lactobacillus*, and *Ruminococcus*, in NAFLD patients compared with healthy controls. Increased abundance of genus *Bacteroides* and decreased *Prevotella* levels have been found in NASH compared with NAFLD patients, and *Ruminococcus* abundance increased in patients in fibrosis stage F ≥ 2 [10]. Furthermore, a random forest model comprising predominantly gut bacterial features showed a strong diagnostic precision to detect advanced fibrosis in NAFLD patients [11,12]. However, most previous studies had a small sample size or inadequately controlled potential confounding factors such as diet and lifestyles, and findings regarding individual taxa associations remain limited and inconsistent [4,6,13].

In the present study, we used resources from two large prospective cohorts of middle-aged to older urban Chinese adults and compared gut microbial diversity and taxonomic composition among over 1500 adults with or without a history of NAFLD. Such comparisons may help better understand the gut–liver axis and identify potentially important gut bacteria that may play a role in NAFLD development and progression, and thus offer innovative options for prevention and treatment of this leading liver disease.

## 2. Materials and Methods

### 2.1. Study Population

Participants of this study were selected from two population-based cohort studies, the Shanghai Women’s Health Study (SWHS) and Shanghai Men’s Health Study (SMHS). The designs and methods of the SWHS and SMHS have been described in detail elsewhere [14,15]. Briefly, the SWHS recruited 74,941 women aged 40–70 years between 1996 and 2000 from urban communities in Shanghai, China, with a response rate of 92.7% [14]. The SMHS recruited 61,480 men aged 40–70 years between 2002 and 2006 from the same communities, with a response rate of 74.0% [15]. In-person interviews were conducted at baseline to collect sociodemographic data, disease history, diet/lifestyles, and anthropometrics; biospecimens were also collected, including blood, urine, and/or oral rinse samples. Participants were followed-up through in-person surveys every 2–4 years (response rates > 92%) with supplemental annual record linkages to Shanghai Vital Statistics and Shanghai Cancer Registry (completion rates > 99%) to collect information on the occurrence of cancer and other chronic diseases including liver diseases, as well as to update information on diet, lifestyle, and anthropometrics. Informed consent was obtained from all study participants. A participant inclusion/exclusion flow chart for the present study is shown in Supplementary Figure S1 and described in detail below.

### 2.2. NAFLD Assessment

Information on fatty liver diagnosis and ultrasound examination was collected during follow-up surveys conducted between 2004 and 2017 (the 3rd to 5th in-person visits of the SWHS and the 2nd and 3rd visits of the SMHS). In each survey, participants were asked whether they had been diagnosed with fatty liver disease by a physician (if yes, the time of diagnosis) and whether there was an abdominal ultrasound. Given that NAFLD is usually asymptomatic, to reduce potential misclassification of NAFLD status, we limited their analysis to participants who had an abdominal ultrasound and answered the fatty liver question. Meanwhile, we only included participants who had no history of viral hepatitis and zero to moderate alcohol consumption (≤1 drink/day for women and ≤2 drinks/day for men; 1 drink = 14 g ethanol), using data from baseline and follow-up surveys. 

### 2.3. Stool Sample Collection and 16S rRNA Gene Sequencing

Stool sample collections were carried out in both cohort studies between 2015 and 2018 (the 5th visit of the SWHS and the 3rd visit of the SMHS). Stool samples were collected from a total of 10,655 participants (5526 women and 5129 men) using the 95% ethanol method, as described in detail in our previous publication [16]. At the time of stool collection, participants were also asked for the date and time of stool collection, antibiotic and medication uses in the past 7 days and 6 months, and whether they had diarrhea in the last 7 days. Stool samples were shipped to the laboratory within 24 h after collection and stored at −80 °C. 

Stool sample DNA of 3358 study participants was isolated using QIAGEN’s DNeasy PowerSoil kit (Germantown, MD, USA). Sequencing libraries were prepared using NEXTflex 16S V4 Amplicon-Seq Kit (Bioo Scientific 4201-05, Austin, TX, USA). The 16S rRNA gene sequencing was performed at pair-end 250 bp using Illumina HiSeq System. For each 96-well plate, one negative control sample (distilled water) was included. The protocols for sequencing data processing and quality controls were published elsewhere [17]. Briefly, raw sequencing data were trimmed and filtered to remove bases and low-quality reads by using Sickle. BayesHammer was utilized to correct sequencing errors and PANDAseq to stitch paired-end reads. Clean reads were then clustered into Operational Taxonomic Units (OTUs) at 97% sequence identity using the closed reference OTU picking strategy, with Greengenes [18] as reference, via the taxonomy classification function “mothur” [19] implemented in Quantitative Insights into Microbial Ecology (QIIME) v1.9.1 [20]. 

As described previously, we obtained 16S rRNA sequencing data from 3194 participants after quality control procedures [16]. Among them, 2358 participants had information on NAFLD history and abdominal ultrasound. For the current study, we further excluded participants who used antibiotics or had diarrhea in the past 7 days before stool collection (*n* = 81) and who were ever diagnosed with or self-reported probably gut microbiome-impacting diseases, including any cancer (*n* = 46), diabetes (*n* = 183), stroke (*n* = 366), or coronary heart disease (*n* = 234) at baseline or during follow-up. A total of 1559 adults, including 759 men and 800 women, were included in the final analysis. 

### 2.4. Statistical Analysis

The analyses were conducted in men and women separately and in a combined dataset adjusting for sex. The sequencing reads per sample ranged between 17,013 and 244,929, with a mean of 134,520. We rarefied the OTU table using the minimal sequencing depth and estimated observed bacterial numbers and α-diversity indices, including Chao1, Shannon, and phylogenetic diversity (PD_whole_tree). A linear regression model was used to evaluate the differences in α-diversity between NAFLD cases and controls. Association between NAFLD and genus level Bray-Curtis β-diversity was evaluated using permutational multivariate analysis of variance (PERMANOVA) with the adonis2 function in R package vegan [21].

The presence of individual taxa was defined as their relative abundance ≥0.00588% in a sample (i.e., ≥1 read when there were 17,013 reads, the minimum sequencing depth of our samples). Common taxa were defined if present in (carrier frequency) >50% of control participants; rare taxa were defined if present in 10–50% of control participants; taxa present in <10% of control participants were excluded from analyses. For common taxa, sequencing counts for each taxon were normalized using centered log-ratio transformation after adding 1 as a pseudo-count [22,23]. General linear regression models were used to evaluate associations of NAFLD with each taxon. Logistic regression was used to evaluate associations between NAFLD and the presence (yes/no) of rare taxa. Potential confounders were adjusted for in two models: the basic model included age at stool collection, sex (for combined analysis), the season of stool collection, education, income, and sequencing batch; the full model further included body mass index (BMI), waist-to-hip ratio (WHR), smoking status, alcohol drinking status, physical activity, total energy intake, dietary fat intake, bowel movement frequency, history of hypertension, and history of dyslipidemia. Associations from the full model were presented as the main results. Sequencing depth was included as an additional covariate for analyses with rare taxa prevalence. Covariates were updated using data from follow-up surveys conducted between 2012 and 2017, except for education and income, which were assessed only at baseline.

Stratified analyses were conducted by age (< or ≥65 years at stool collection), overweight (BMI < or ≥24 kg/m^2^, according to recommendation for Chinese adults [24]), WHR (men: < or ≥0.9; women: < or ≥0.8), healthy diet score (< or ≥24.5 [median]), history of dyslipidemia, history of hypertension, and time between self-reported NAFLD diagnosis and stool collection (< or ≥9.5 years [median]; or <5, 5–15, or ≥15 years). An interaction term of NAFLD with a stratified variable was added to the regression model. The Benjamini-Hochberg false discovery rate (FDR) was applied to account for multiple comparisons at each taxonomic level. Significance was defined at an FDR < 0.1 at each taxonomic level. All analyses were carried out using QIIME [20], SAS Enterprise Guide 7.1 (SAS Institute Inc., Cary, NC, USA), or R version 3.6.3.

## 3. Results

### 3.1. Characteristics of the Study Subjects

The current study included 188 men and 233 women with NAFLD and 571 men and 567 women without NAFLD. Compared with non-NAFLD controls, participants with NAFLD had higher BMI (mean: 25.8 vs. 23.9 among men; 26.1 vs. 23.5 among women), WHR (mean: 0.92 vs. 0.89 among men; 0.83 vs. 0.81 among women), and prevalence of dyslipidemia (19.2% vs. 6.0% among men; 44.6% vs. 14.6% among women) (Table 1; all *p* < 0.001). Meanwhile, female cases had a higher income level, lower dietary fat intake, and higher prevalence of hypertension. Otherwise, participants with or without a history of NAFLD did not differ by age (mean: 68 years at stool collection; range: 51–89 years), education level, smoking status, alcohol drinking status, overall diet quality, total energy intake, and bowel movement frequency.

### 3.2. Associations of NAFLD History with Gut Microbiome Alpha and Beta Diversity

**Figure 1:**
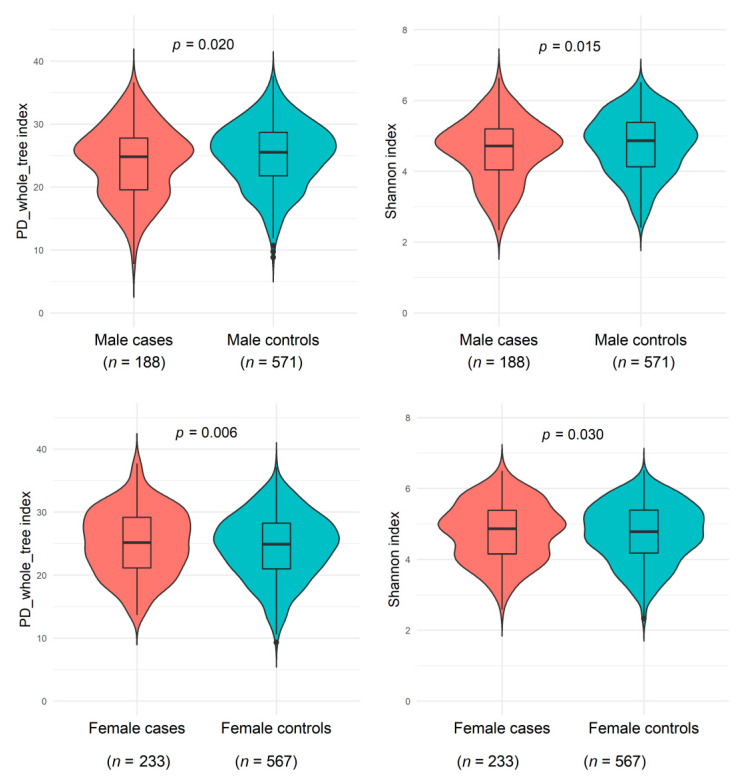
Gut microbiome α-diversity indexes (PD_whole_tree_distance and Shannon) between non-alcoholic fatty liver disease and healthy controls among males from the Shanghai Men’s Health Study (SMHS) and females from the Shanghai Women’s Health Study (SWHS). General linear regression was conducted, adjusting for age at stool collection, the season of stool collection, body mass index, waist-to-hip ratio, education, income, smoking status, alcohol drinking status, physical activity, total energy intake, fat intake, bowel movement frequency, history of hypertension, history of dyslipidemia, and sequencing batch. Abbreviation: PD, phylogenetic diversity.

As shown in Figure 1 and Supplementary Table S1, men with a NAFLD history had slightly decreased microbiome α-diversity (including PD_whole_tree, Shannon index, Chao1, and observed OTUs) than men without a history of NAFLD, whereas women with NAFLD showed slightly increased α-diversity than women without NAFLD (all *p* < 0.05 compared with controls). A potential effect modification by sex on the NAFLD and α-diversity association was suggested (all *p* < 0.02 for interactions). The genus-level Bray-Curtis dissimilarities between NAFLD cases and controls were not significant in either sex; NAFLD status explained 0.23% and 0.09% Bray-Curtis variance among men and women, respectively.

### 3.3. Associations of NAFLD History with Individual Gut Microbial Taxa

Similar to the α-diversity results, we observed significant sex-specific associations between NAFLD history and individual taxa (Table 2). We examined 145 common taxa (5 phyla, 10 classes, 12 orders, 20 families, 38 genera, and 60 species). Among men, NAFLD was associated with increased abundance of genera *Dialister* (median relative abundance: 0.0554% in cases vs. 0.0214% in controls; *p* = 0.001) and *Streptococcus* (0.1144% vs. 0.0787%; *p* = 0.01), two *Bifidobacterium* species (both *p* = 0.03 for *B. adolescentis* and *B. Other*), and an unclassified *Dialister* species, while a decreased abundance of genus *Phascolarctobacterium* (0.9446% vs. 1.672%; *p* = 0.01). Among women, NAFLD was associated with increased abundance of genus *Butyricimonas* (0.1061% vs. 0.0463%; *p* = 0.003) and an unclassified species within it, an unclassified family and genus of order *Clostridiales* (0.0127% vs. 0.0052%; *p* = 0.003), and an *Oscillospira* species (0.0275% vs. 0.0146%; *p* = 0.009). Significant interactions between NAFLD history and sex were observed for all these associations (all FDR < 0.1 for interactions). In the combined dataset with additional adjustment for sex, the abundance of an unclassified *Streptococcus* species was higher, while the abundance of an unclassified Blautia species was lower in NAFLD cases than controls (all *p* < 0.05, Supplementary Table S2).

Among 152 rare taxa (5 phyla, 7 classes, 8 orders, 23 families, 44 genera, and 65 species), 18 showed significant opposite associations with NAFLD between men and women (Table 3, all NAFLD–sex interaction FDR < 0.1). NAFLD was generally associated with decreased taxa prevalence in men and increased prevalence in women, including unclassified genus and species of a proposed family (*Mogibacteriaceae*) (in men: carriage frequency: 32.4% in cases vs. 45.9% in controls; *p* ≤ 0.005), a species under family *Rikenellaceae* (41% vs. 50.3% in men; 49.8% vs. 43.7% in women; both *p* < 0.05), an unclassified genus and species of family *Peptococcaceae* (10.6% vs. 20.3%; both *p* = 0.04 in men), an unclassified genus and species of family *Christensenellaceae* (36.1% vs. 31.9%; both *p* = 0.008 in women), and order *RF39* and an unclassified genus and species within it (16.5% vs. 25.9% in men; 27% vs. 21.5% in women; all *p* < 0.05). In the combined dataset, species *Coprococcus eutactus* and genus *Megasphaera* were more prevalent in NAFLD cases than controls (Supplementary Table S3). A higher prevalence of *Megasphaera* in NAFLD cases than controls was observed among all participants (30.2% vs. 22.4%; *p* = 5.7 × 10^−4^, FDR = 0.047) and in both men (31.4% vs. 24.6%; *p* = 0.017) and women (29.2% vs. 21.2%; *p* = 0.006).

We evaluated further the sex-specific NAFLD–microbiome associations by age, BMI, WHR, healthy diet score, and history of hypertension (Supplementary Table S4). In men, we found significantly increased abundance of *Bifidobacterium* species in those <65 years with NAFLD (median relative abundance: 0.0893% vs. 0.0384% for *B. adolescentis*, *p* = 3.6 × 10^−4^; 0.0196% vs. 0.0073% for *B. Other*, *p* ≤ 1.6 × 10^−4^) but not in those 65 years or older (0.0308% vs. 0.0475% for *B. adolescentis*, *p* = 0.575; 0.0046% vs. 0.0080% for *B. Other*, *p* = 0.441; *p* ≤ 4.9 × 10^−4^ for NAFLD–age interaction); significantly higher frequency of *Blautia producta* in male NAFLD cases with WHR< 0.9 (41% vs. 21.6%, *p* = 0.003) while slightly lower in cases with WHR ≥ 0.9 (18.9% vs. 27.8%, *p* = 0.151; *p* ≤ 2.2 × 10^−4^ for NAFLD–WHR interaction), and significantly lower prevalence of order *RF39* in NAFLD cases without a history of hypertension (14.3% vs. 28%, *p* = 0.003) but increased prevalence of this order in 34 male NAFLD cases with hypertension (26.5% vs. 12.7%, *p* = 0.105; *p* = 0.005 for NAFLD– hypertension interaction). We did not find significant differences in the sex-specific NAFLD–microbiome associations by history of dyslipidemia or time interval between NAFLD diagnosis and stool collection (all FDR > 0.1 for NAFLD–dyslipidemia interaction or NAFLD–time interaction). In addition, most of the associations from the full model were similar to those from the basic model, which included age, sex, education, income, sample collection season, and sequencing batch.

## 4. Discussion

In this study of 1559 predominantly elderly urban Chinese adults, we found that NAFLD was associated with gut microbiome α-diversity and several taxa differently in men and women, suggesting the importance of considering sex/gender in research of the gut–liver axis. Among men, NAFLD was associated with decreased microbial α-diversity, increased abundance of genera *Dialister* and *Streptococcus* and *Bifidobacterium* species, reduced abundance of genus *Phascolarctobacterium*, and reduced prevalence of order *RF39* and unclassified genus/species of families (*Mogibacteriaceae*), *Rikenellaceae*, and *Peptococcaceae*. In contrast, among women, NAFLD was associated with increased microbial α-diversity and altered abundance and prevalence of above taxa, generally in the opposite direction. We also found that age, BMI, WHR, diet quality, and history of hypertension may modify NAFLD association with specific taxa in men or women.

Increasing evidence supports sex differences in the gut microbiome and potential sex-dependent associations of gut microbiota with health outcomes [25,26,27,28,29]. In the present study, we observed significant associations of NAFLD with microbial α-diversity and individual taxa varied by sex, but no significant sex differences in those microbial features (i.e., similar diversity and abundance/prevalence between men and women among NAFLD cases or controls). The underlying mechanisms for such findings are not clear, although sex differences in the gut microbiome, hormone, BMI, and lifestyles have been shown [27]. The observed sex-specific associations might be due to older age at stool collection in women than in men [30,31,32], and much fewer smokers and alcohol drinkers, high prevalence of morbidities such as hypertension and dyslipidemia, or changes in diet and lifestyles after disease diagnosis among women than men; however, all those covariates had been adjusted for in our main models. Future studies are needed to examine the sex-specific gut microbiome associations with NAFLD and investigate underlying biological mechanisms. 

Previous studies have shown altered gut microbiota in NAFLD cases compared with controls; however, most of these studies comprised only ~20–50 NAFLD cases and controls and included limited dietary, lifestyle, and clinical factors [10,11,12,33,34,35,36,37,38,39,40,41,42,43,44]. Still, a few NAFLD–microbiome associations have been suggested [4,6,13]. In line with previous studies [12,36,38,39,40,42], we observed increased abundance of genus *Streptococcus* in NAFLD cases among men and in the combined dataset (*p* = 0.015 and 0.022, respectively). In addition to NAFLD, several *Streptococcus* species have been associated with inflammatory bowel disease [45,46,47], suggesting a pro-inflammatory role of *Streptococcus* in gut–liver axis-related diseases. Previous studies have also linked NAFLD with increased abundance of genus *Escherichia*, another pro-inflammatory bacterium that may produce ethanol [36,38,40,44], and decreased abundance of genera *Ruminococcus* and *Coprococcus* under order *Clostridiales*, which are fiber-fermenting, SCFA-generating bacteria [4], but significant associations were not observed for those genera among men or women. Nevertheless, we found Blautia, also a fiber-fermenting, SCFA-generating genus under order *Clostridiales*, showing a decreased abundance in NAFLD cases among total participants. Meanwhile, consistent with prior work showing a reduced abundance of genus *Bifidobacterium* in NASH patients, we observed reduced abundance of *Bifidobacterium* in female NAFLD cases (median RA: 0.25% vs. 0.34%, *p* = 0.039) (Table 2). Further studies are warranted to evaluate the relationships of gut inflammation and bacterial production of ethanol and SCFAs with NAFLD.

In addition, we found a significantly increased abundance of genus *Dialister* in male NAFLD cases but decreased level in female cases. *Dialister* is a genus of *Firmicutes*, which was found to increase among liver cirrhosis patients [48,49]. Genus *Phascolarctobacterium* has been associated with age and weight loss in NAFLD patients [50]. Its abundance has also shown sex-difference in metabolic syndrome patients, i.e., higher in female than in male patients [51]. We observed a reduced *Phascolarctobacterium* abundance among male NAFLD cases but an increased abundance among female NAFLD cases. Among rare taxa that showed sex-dependent associations with NAFLD, order *RF39* has been positively associated with healthy diets [16,52] and negatively associated with BMI, blood triglycerides, and frailty among older adults [52,53,54,55,56], suggesting its potential health benefits. 

This study has several strengths. First, this is the largest population-based study to date identifying gut microbiome features related to history of NAFLD in an Asian population. Second, information is available on a wide range of medical, sociodemographic, and lifestyle factors, allowing us to exclude participants with a history of other diseases (e.g., diabetes, cardiovascular disease, and hepatitis) and adjust for various covariates to minimize potential confounding. Third, for the first time, we found potential sex-dependent gut microbial features related to NAFLD, although further studies are needed to validate such findings. Several limitations should also be acknowledged. First, there may be misclassification of NAFLD status, which may attenuate the observed associations. Second, despite comprehensive covariate adjustments, the impact of residual confounding due to poorly measured or unmeasured variables such as other underlying diseases and medication uses cannot be overlooked [57,58,59]. At the same time, some variables included in the full model may be confounders and were also involved in the causal pathways between NAFLD and gut microbiota (e.g., WHR and history of dyslipidemia). However, a minimal adjustment model yielded similar results to the full model. Third, stool samples were collected 2.2 to 35.3 years (median 9.5 years) after the first NAFLD diagnosis, while we did not know how the disease may have progressed during this time. However, we did not find significant effect modifications by time period: the NAFLD–time interaction was not significant; the sex-specific associations presented in all tables generally remained when we limited NAFLD cases to those diagnosed <15 years before stool collection (336 cases), and association directions were consistent when limited to diagnosed <5 years (*n* = 68) or ≥15 years (*n* = 85) before stool collection. Fourth, stool samples were stored at −80 °C for up to three years before sequencing. Although recent studies showed that long-term storage at −80 °C (i.e., up to five years) has limited effects on 16S rRNA sequencing results of human fecal samples [60,61], we do not know how the sample storage may have affected our results, particularly for rare or low-abundance taxa. Finally, due to the bidirectional relation between gut microbiota and NAFLD [62], how the observed microbiota alterations may affect NAFLD development or progression is unclear and needs to be clarified in future studies.

In summary, in a large cohort of older, urban Chinese adults, we found significant sex-specific associations of NAFLD history with gut microbiome α-diversity and composition. Further studies are needed to validate these findings and investigate whether those gut microbial changes may play a role in the development or progression of NAFLD. 

## Figures and Tables

**Table 1:** Selected characteristics of participants from the Shanghai Men’s and Women’s Health Study.

Characteristics	Shanghai Men’s Health Study			Shanghai Women’s Health Study		
	Controls (*n* = 571)	NAFLD (*n* = 188)	*p* ^a^	Controls (*n* = 567)	NAFLD (*n* = 233)	*p* ^a^
Age at stool sample collection (years, mean (SD))	67.9 (9.4)	66.7 (8.2)	0.189	69.9 (8.8)	69.7 (7.4)	0.750
Recent body mass index (kg/m^2^, mean (SD))	23.9 (3.2)	25.8 (3.1)	<0.001	23.5 (3.6)	26.1 (3.6)	<0.001
Recent waist-to-hip ratio (mean (SD))	0.89 (0.06)	0.92 (0.05)	<0.001	0.81 (0.05)	0.83 (0.05)	<0.001
Education (%)			0.280			0.171
Elementary school or less	5.6	4.3		17.8	12.0	
Middle school	29.6	36.7		41.5	41.6	
High school graduate	37.7	32.4		28.0	30.5	
Some college and higher	27.1	26.6		12.7	15.9	
High income (%)	8.8	10.6	0.096	15.9	22.3	0.020
Smoking						
Never smoker (%)	39.9	35.6	0.248	99.1	99.6	0.500
Former smoker (%)	13.0	17.6		0.9	0.4	
Current smoker (%)	47.1	46.8				
Pack-year among smokers (mean (SD))	24.2 (14.9)	23.8 (15.4)	0.815	-	-	-
Alcohol drinking						
Ever alcohol drinking (%)	34.7	29.8	0.218	4.4	7.3	0.096
Alcohol consumption (g/day, mean (SD)) ^b^	2.28 (1.82)	2.65 (2.32)	0.188	0.13 (0.54)	0.07 (0.13)	0.110
Stool sample collection season (%)			0.602			0.273
Spring	10	13.3		10.4	15	
Summer	6.8	6.9		7.4	8.2	
Autumn	36.4	36.7		41.1	39.9	
Winter	46.8	43.1		41.1	36.9	
Leisure-time physical activity (yes, %)	55.7	53.2	0.550	61.9	56.2	0.136
Healthy diet score (mean (SD))	25.1 (5.1)	25.2 (5.5)	0.655	24.1 (5.0)	23.9 (5.0)	0.651
Total energy intake (kcal/day, mean (SD))	1968 (488)	1954 (460)	0.994	1508 (307)	1550 (338)	0.079
Fiber intake (g/day, mean (SD))	12.3 (3.6)	12.1 (3.5)	0.508	10.0 (2.7)	10.1 (2.4)	0.362
Fat intake (g/day, mean (SD))	33.9 (10.0)	34.3 (10.4)	0.986	28.5 (7.1)	27.3 (7.1)	0.006
History of hypertension (yes, %)	13.8	18.1	0.156	18.7	26.6	0.013
History of dyslipidemia (yes, %)	6.0	19.2	<0.001	14.6	44.6	<0.001
Bowel movement (times/week, mean (SD))	7.7 (3.6)	7.8 (3.6)	0.926	7.3 (3.7)	7.7 (4.0)	0.071

^a^ Wilcoxon rank sum tests for continuous variables and chi-square test for categorical variables. ^b^ Among alcohol drinkers. NAFLD, non-alcoholic fatty liver disease; SD, standard deviation.

**Table 2:** Sex-dependent association of non-alcoholic fatty liver disease and common bacterial taxa ^a^.

	SMHS				SWHS				*p* for Interaction with Sex ^d^
	Non-NAFLD (*n* = 571)	NAFLD (*n* = 188)			Non-NAFLD (*n* = 567)	NAFLD (*n* = 233)			
Taxon ^b^	Median RA (%)	Median RA (%)	Beta (se) ^c^	*p* ^c^	Median RA (%)	Median RA (%)	Beta (se) ^c^	*p* ^c^	
p_*Actinobacteria*;c_*Actinobacteria*;o_*Bifidobacteriales*;f_*Bifidobacteriaceae*;g_*Bifidobacterium*;*Other*	0.0075	0.0106	0.369 (0.171)	0.031	0.0089	0.0068	−0.439 (0.163)	0.007	0.002
p_*Actinobacteria*;c_*Actinobacteria*;o_*Bifidobacteriales*;f_*Bifidobacteriaceae*;g_*Bifidobacterium*;s_*adolescentis*	0.0441	0.0585	0.474 (0.218)	0.03	0.0635	0.0506	−0.399 (0.207)	0.055	0.008
p_*Bacteroidetes*;c_*Bacteroidia*;o_*Bacteroidales*;f_[*Odoribacteraceae*];g_*Butyricimonas*	0.0906	0.032	−0.238 (0.221)	0.28	0.0463	0.1061	0.632 (0.214)	0.003	0.007
p_*Bacteroidetes*;c_*Bacteroidia*;o_*Bacteroidales*;f_[*Odoribacteraceae*];g_*Butyricimonas*;s_*unclassified*	0.0906	0.032	−0.23 (0.217)	0.291	0.0463	0.1061	0.639 (0.212)	0.003	0.005
p_*Firmicutes*;c_*Bacilli*;o_*Lactobacillales*;f_*Streptococcaceae*;g_*Streptococcus*	0.0787	0.1144	0.363 (0.148)	0.015	0.1027	0.0804	−0.104 (0.139)	0.454	0.012
p_*Firmicutes*;c_*Clostridia*;o_*Clostridiales*;f_*unclassified*	0.0113	0.0043	−0.277 (0.231)	0.231	0.0052	0.0127	0.674 (0.225)	0.003	0.002
p_*Firmicutes*;c_*Clostridia*;o_*Clostridiales*;f_*unclassified*;g_*unclassified*	0.0113	0.0043	−0.239 (0.228)	0.294	0.0052	0.0127	0.66 (0.222)	0.003	0.002
p_*Firmicutes*;c_*Clostridia*;o_*Clostridiales*;f_*unclassified*;g_*unclassified*;s_*unclassified*	0.0113	0.0043	−0.23 (0.228)	0.313	0.0052	0.0127	0.667 (0.222)	0.003	0.002
p_*Firmicutes*;c_*Clostridia*;o_*Clostridiales*;f_*Ruminococcaceae*;g_*Oscillospira*;*Other*	0.0425	0.0093	−0.307 (0.195)	0.115	0.0146	0.0275	0.477 (0.183)	0.009	0.005
p_*Firmicutes*;c_*Clostridia*;o_*Clostridiales*;f_*Veillonellaceae*;g_*Dialister*	0.0214	0.0554	0.788 (0.241)	0.001	0.0467	0.0305	−0.487 (0.241)	0.044	2.4 × 10^−4^
p_*Firmicutes*;c_*Clostridia*;o_*Clostridiales*;f_*Veillonellaceae*;g_*Dialister*;s_*unclassified*	0.0214	0.0554	0.796 (0.243)	0.001	0.0467	0.0305	−0.481 (0.245)	0.05	3.3 × 10^−4^
p_*Firmicutes*;c_*Clostridia*;o_*Clostridiales*;f_*Veillonellaceae*;g_*Phascolarctobacterium*	1.672	0.9446	−0.555 (0.217)	0.011	1.4603	1.6876	0.376 (0.238)	0.116	0.007
p_*Firmicutes*;c_*Clostridia*;o_*Clostridiales*;f_*Veillonellaceae*;g_*Phascolarctobacterium*;s_*unclassified*	1.672	0.9446	−0.546 (0.216)	0.012	1.4603	1.6876	0.382 (0.238)	0.108	0.006

^a^ The common taxa were defined as those with relative abundance ≥0.00588% and present in (carrier frequency) >50% of control participants. ^b^ p_, c_, o_, f_, g_, and s_ indicate taxonomic levels of phylum, class, order, family, genus, and species, respectively. ^c^ For each sample, centered log-ratio transformation was used to normalize taxa counts at each taxonomic level after adding a pseudo-count of 1. Beta, se and *p* values were calculated from general linear regression with NAFLD controls as reference, adjusted for age at stool sampling, the season of sample collection, body mass index, waist-to-hip ratio, education, income, smoking status, alcohol drinking status, physical activity, total energy intake, fat intake, bowel movement frequency, history of hypertension, history of dyslipidemia, and sequencing batch. ^d^ False discovery rate < 0.1 at each taxonomic level. NAFLD, non-alcoholic fatty liver disease; RA, relative abundance; se, standard error; SMHS, Shanghai Men’s Health Study; SWHS, Shanghai Women’s Health Study.

**Table 3:** Sex-dependent associations of non-alcoholic fatty liver disease and rare bacterial taxa ^a^.

	SMHS				SWHS				*p* for Interaction with Sex ^d^
	Non-NAFLD (*n* = 571)	NAFLD (*n* = 188)			Non-NAFLD (*n* = 567)	NAFLD (*n* = 233)			
Taxon ^b^	Carrier Frequency (%)	Carrier Frequency (%)	Beta (se) ^c^	*p* ^c^	Carrier Frequency (%)	Carrier Frequency (%)	Beta (se) ^c^	*p* ^c^	
p_*Bacteroidetes*;c_*Bacteroidia*;o_*Bacteroidales*;f_[*Paraprevotellaceae*]	50.8	42	−0.314 (0.189)	0.097	39.3	47.6	0.384 (0.182)	0.035	0.008
p_*Bacteroidetes*;c_*Bacteroidia*;o_*Bacteroidales*;f_*Rikenellaceae*;f\_\_*Rikenellaceae*_*unclassified*	50.3	41	−0.421 (0.187)	0.024	43.7	49.8	0.400 (0.182)	0.028	0.005
p_*Bacteroidetes*;c_*Bacteroidia*;o_*Bacteroidales*;f_*Rikenellaceae*;f\_\_*Rikenellaceae*_*unclassified*;*Other*	50.3	41	−0.421 (0.187)	0.024	43.7	49.8	0.400 (0.182)	0.028	0.005
p_*Firmicutes*;c_*Clostridia*;o_*Clostridiales*;f_[*Mogibacteriaceae*]	51.3	37.8	−0.551 (0.191)	0.004	48.7	49.8	0.285 (0.186)	0.125	0.002
p_*Firmicutes*;c_*Clostridia*;o_*Clostridiales*;f_[*Mogibacteriaceae*];g_*unclassified*	45.9	32.4	−0.551 (0.197)	0.005	42.7	44.2	0.344 (0.186)	0.064	0.001
p_*Firmicutes*;c_*Clostridia*;o_*Clostridiales*;f_[*Mogibacteriaceae*];g_*unclassified*;s_*unclassified*	45.9	32.4	−0.551 (0.197)	0.005	42.7	44.2	0.344 (0.186)	0.064	0.001
p_*Firmicutes*;c_*Clostridia*;o_*Clostridiales*;f_*Christensenellaceae*	35.2	26.6	−0.312 (0.207)	0.132	34.4	36.9	0.422 (0.193)	0.029	0.007
p_*Firmicutes*;c_*Clostridia*;o_*Clostridiales*;f_*Christensenellaceae*;g_*unclassified*	32.7	25.5	−0.300 (0.210)	0.154	31.9	36.1	0.516 (0.196)	0.008	0.005
p_*Firmicutes*;c_*Clostridia*;o_*Clostridiales*;f_*Christensenellaceae*;g_*unclassified*;s_*unclassified*	32.7	25.5	−0.300 (0.210)	0.154	31.9	36.1	0.516 (0.196)	0.008	0.005
p_*Firmicutes*;c_*Clostridia*;o_*Clostridiales*;f_*Peptococcaceae*	24.9	16	−0.481 (0.240)	0.045	24	25.3	0.256 (0.212)	0.227	0.011
p_*Firmicutes*;c_*Clostridia*;o_*Clostridiales*;f_*Peptococcaceae*;g_*unclassified*	20.3	10.6	−0.572 (0.278)	0.040	19.6	21.5	0.352 (0.226)	0.120	0.002
p_*Firmicutes*;c_*Clostridia*;o_*Clostridiales*;f_*Peptococcaceae*;g_*unclassified*;s_*unclassified*	20.3	10.6	−0.572 (0.278)	0.040	19.6	21.5	0.352 (0.226)	0.120	0.002
p_*Tenericutes*	28.2	20.2	−0.323 (0.224)	0.149	24	27.5	0.327 (0.207)	0.115	0.004
p_*Tenericutes*;c_*Mollicutes*	26.6	17	−0.517 (0.234)	0.027	21.5	27	0.417 (0.211)	0.049	3.6 × 10^−4^
p_*Tenericutes*;c_*Mollicutes*;o_*RF39*	25.9	16.5	−0.516 (0.237)	0.029	21.5	27	0.417 (0.211)	0.049	3.8 × 10^−4^
p_*Tenericutes*;c_*Mollicutes*;o_*RF39*;f_*unclassified*	25.9	16.5	−0.516 (0.237)	0.029	21.5	27	0.417 (0.211)	0.049	3.8 × 10^−4^
p_*Tenericutes*;c_*Mollicutes*;o_*RF39*;f_*unclassified*;g_*unclassified*	25.9	16.5	−0.516 (0.237)	0.029	21.5	27	0.417 (0.211)	0.049	3.8 × 10^−4^
p_*Tenericutes*;c_*Mollicutes*;o_*RF39*;f_*unclassified*;g_*unclassified*;s_*unclassified*	25.9	16.5	−0.516 (0.237)	0.029	21.5	27	0.417 (0.211)	0.049	3.8 × 10^−4^

^a^ The rare taxa were defined as those with relative abundance ≥0.00588% and present in (carrier frequency) 10–50% of control participants. ^b^ p_, c_, o_, f_, g_, and s_ indicate taxonomic levels of phylum, class, order, family, genus, and species, respectively. ^c^ Logistic regression model for NAFLD association with rare taxa, adjusted for age at stool sampling, the season of sample collection, body mass index, waist-to-hip ratio, education, income, smoking status, alcohol drinking status, physical activity, total energy intake, fat intake, bowel movement frequency, history of hypertension, history of dyslipidemia, sequencing batch, and sequencing depth. ^d^ False discovery rate (FDR) < 0.1 at each taxonomic level. NAFLD, non-alcoholic fatty liver disease; RA, relative abundance; se, standard error; SMHS, Shanghai Men’s Health Study; SWHS, Shanghai Women’s Health Study.

## Data Availability

Data used in the present study can be requested from the corresponding author.

## Supplementary Materials

The following are available online at https://www.mdpi.com/article/10.3390/microorganisms9102118/s1, Supplementary Figure S1: Flow chart of study participants inclusion and exclusion, Supplementary Table S1: Association of no-alcoholic fatty liver disease and gut microbial richness and a-diversity metrics, Supplementary Table S2: Nominal association of non-alcoholic fatty liver disease and common bacterial taxa, Supplementary Table S3: Nominal association of non-alcoholic fatty liver disease and rare gut bacterial taxa, Supplementary Table S4: Significant interactions between NAFLD and non-sex categorical variables with individual taxa associations.

## References

[1] Yki-Järvinen H. (2014). Non-Alcoholic Fatty Liver Disease as a Cause and a Consequence of Metabolic Syndrome. Lancet Diabetes Endocrinol..

[2] Younossi Z.M. (2019). Non-Alcoholic Fatty Liver Disease—A Global Public Health Perspective. J. Hepatol..

[3] Zhou F., Zhou J., Wang W., Zhang X.-J., Ji Y.-X., Zhang P., She Z.-G., Zhu L., Cai J., Li H. (2019). Unexpected Rapid Increase in the Burden of NAFLD in China From 2008 to 2018: A Systematic Review and Meta-Analysis. Hepatology.

[4] Safari Z., Gérard P. (2019). The Links between the Gut Microbiome and Non-Alcoholic Fatty Liver Disease (NAFLD). Cell. Mol. Life Sci. CMLS.

[5] Albillos A., de Gottardi A., Rescigno M. (2020). The Gut-Liver Axis in Liver Disease: Pathophysiological Basis for Therapy. J. Hepatol..

[6] Grabherr F., Grander C., Effenberger M., Adolph T.E., Tilg H. (2019). Gut Dysfunction and Non-Alcoholic Fatty Liver Disease. Front. Endocrinol..

[7] Huang T.D., Behary J., Zekry A. (2020). Non-Alcoholic Fatty Liver Disease: A Review of Epidemiology, Risk Factors, Diagnosis and Management. Intern. Med. J..

[8] Le Roy T., Llopis M., Lepage P., Bruneau A., Rabot S., Bevilacqua C., Martin P., Philippe C., Walker F., Bado A. (2013). Intestinal Microbiota Determines Development of Non-Alcoholic Fatty Liver Disease in Mice. Gut.

[9] Henao-Mejia J., Elinav E., Jin C., Hao L., Mehal W.Z., Strowig T., Thaiss C.A., Kau A.L., Eisenbarth S.C., Jurczak M.J. (2012). Inflammasome-Mediated Dysbiosis Regulates Progression of NAFLD and Obesity. Nature.

[10] Boursier J., Mueller O., Barret M., Machado M., Fizanne L., Araujo-Perez F., Guy C.D., Seed P.C., Rawls J.F., David L.A. (2016). The Severity of Nonalcoholic Fatty Liver Disease Is Associated with Gut Dysbiosis and Shift in the Metabolic Function of the Gut Microbiota. Hepatology.

[11] Loomba R., Seguritan V., Li W., Long T., Klitgord N., Bhatt A., Dulai P.S., Caussy C., Bettencourt R., Highlander S.K. (2017). Gut Microbiome-Based Metagenomic Signature for Non-Invasive Detection of Advanced Fibrosis in Human Nonalcoholic Fatty Liver Disease. Cell Metab..

[12] Caussy C., Tripathi A., Humphrey G., Bassirian S., Singh S., Faulkner C., Bettencourt R., Rizo E., Richards L., Xu Z.Z. (2019). A Gut Microbiome Signature for Cirrhosis Due to Nonalcoholic Fatty Liver Disease. Nat. Commun..

[13] Li F., Ye J., Shao C., Zhong B. (2021). Compositional Alterations of Gut Microbiota in Nonalcoholic Fatty Liver Disease Patients: A Systematic Review and Meta-Analysis. Lipids Health Dis..

[14] Zheng W., Chow W.-H., Yang G., Jin F., Rothman N., Blair A., Li H.-L., Wen W., Ji B.-T., Li Q. (2005). The Shanghai Women’s Health Study: Rationale, Study Design, and Baseline Characteristics. Am. J. Epidemiol..

[15] Shu X.-O., Li H., Yang G., Gao J., Cai H., Takata Y., Zheng W., Xiang Y.-B. (2015). Cohort Profile: The Shanghai Men’s Health Study. Int. J. Epidemiol..

[16] Yu D., Nguyen S.M., Yang Y., Xu W., Cai H., Wu J., Cai Q., Long J., Zheng W., Shu X.-O. (2021). Long-Term Diet Quality Is Associated with Gut Microbiome Diversity and Composition among Urban Chinese Adults. Am. J. Clin. Nutr..

[17] Yang Y., Cai Q., Shu X.-O., Steinwandel M.D., Blot W.J., Zheng W., Long J. (2019). Prospective Study of Oral Microbiome and Colorectal Cancer Risk in Low-Income and African American Populations. Int. J. Cancer.

[18] McDonald D., Price M.N., Goodrich J., Nawrocki E.P., DeSantis T.Z., Probst A., Andersen G.L., Knight R., Hugenholtz P. (2012). An Improved Greengenes Taxonomy with Explicit Ranks for Ecological and Evolutionary Analyses of Bacteria and Archaea. ISME J..

[19] Schloss P.D., Westcott S.L., Ryabin T., Hall J.R., Hartmann M., Hollister E.B., Lesniewski R.A., Oakley B.B., Parks D.H., Robinson C.J. (2009). Introducing Mothur: Open-Source, Platform-Independent, Community-Supported Software for Describing and Comparing Microbial Communities. Appl. Environ. Microbiol..

[20] Caporaso J.G., Kuczynski J., Stombaugh J., Bittinger K., Bushman F.D., Costello E.K., Fierer N., Peña A.G., Goodrich J.K., Gordon J.I. (2010). QIIME Allows Analysis of High-Throughput Community Sequencing Data. Nat. Methods.

[21] Dixon P. (2003). VEGAN, a Package of R Functions for Community Ecology. J. Veg. Sci..

[22] Fernandes A.D., Reid J.N., Macklaim J.M., McMurrough T.A., Edgell D.R., Gloor G.B. (2014). Unifying the Analysis of High-Throughput Sequencing Datasets: Characterizing RNA-Seq, 16S RRNA Gene Sequencing and Selective Growth Experiments by Compositional Data Analysis. Microbiome.

[23] Knight R., Vrbanac A., Taylor B.C., Aksenov A., Callewaert C., Debelius J., Gonzalez A., Kosciolek T., McCall L.-I., McDonald D. (2018). Best Practices for Analysing Microbiomes. Nat. Rev. Microbiol..

[24] Zhou B.-F. (2002). Predictive Values of Body Mass Index and Waist Circumference for Risk Factors of Certain Related Diseases in Chinese Adults--Study on Optimal Cut-off Points of Body Mass Index and Waist Circumference in Chinese Adults. Biomed. Environ. Sci. BES.

[25] de la Cuesta-Zuluaga J., Kelley S.T., Chen Y., Escobar J.S., Mueller N.T., Ley R.E., McDonald D., Huang S., Swafford A.D., Knight R. (2019). Age- and Sex-Dependent Patterns of Gut Microbial Diversity in Human Adults. mSystems.

[26] Sinha T., Vich Vila A., Garmaeva S., Jankipersadsing S.A., Imhann F., Collij V., Bonder M.J., Jiang X., Gurry T., Alm E.J. (2019). Analysis of 1135 Gut Metagenomes Identifies Sex-Specific Resistome Profiles. Gut Microbes.

[27] Kim Y.S., Unno T., Kim B.Y., Park M.S. (2020). Sex Differences in Gut Microbiota. World J. Mens Health.

[28] Peterson V.L., Richards J.B., Meyer P.J., Cabrera-Rubio R., Tripi J.A., King C.P., Polesskaya O., Baud A., Chitre A.S., Bastiaanssen T.F.S. (2020). Sex-Dependent Associations between Addiction-Related Behaviors and the Microbiome in Outbred Rats. EBioMedicine.

[29] Yuan X., Chen R., Zhang Y., Lin X., Yang X. (2020). Sexual Dimorphism of Gut Microbiota at Different Pubertal Status. Microb. Cell Factories.

[30] Yatsunenko T., Rey F.E., Manary M.J., Trehan I., Dominguez-Bello M.G., Contreras M., Magris M., Hidalgo G., Baldassano R.N., Anokhin A.P. (2012). Human Gut Microbiome Viewed across Age and Geography. Nature.

[31] Lakshminarayanan B., Stanton C., O’Toole P.W., Ross R.P. (2014). Compositional Dynamics of the Human Intestinal Microbiota with Aging: Implications for Health. J. Nutr. Health Aging.

[32] Odamaki T., Kato K., Sugahara H., Hashikura N., Takahashi S., Xiao J.-Z., Abe F., Osawa R. (2016). Age-Related Changes in Gut Microbiota Composition from Newborn to Centenarian: A Cross-Sectional Study. BMC Microbiol..

[33] Raman M., Ahmed I., Gillevet P.M., Probert C.S., Ratcliffe N.M., Smith S., Greenwood R., Sikaroodi M., Lam V., Crotty P. (2013). Fecal Microbiome and Volatile Organic Compound Metabolome in Obese Humans with Nonalcoholic Fatty Liver Disease. Clin. Gastroenterol. Hepatol. Off. Clin. Pract. J. Am. Gastroenterol. Assoc..

[34] Wong V.W.-S., Tse C.-H., Lam T.T.-Y., Wong G.L.-H., Chim A.M.-L., Chu W.C.-W., Yeung D.K.-W., Law P.T.-W., Kwan H.-S., Yu J. (2013). Molecular Characterization of the Fecal Microbiota in Patients with Nonalcoholic Steatohepatitis—A Longitudinal Study. PLoS ONE.

[35] Zhu L., Baker S.S., Gill C., Liu W., Alkhouri R., Baker R.D., Gill S.R. (2013). Characterization of Gut Microbiomes in Nonalcoholic Steatohepatitis (NASH) Patients: A Connection between Endogenous Alcohol and NASH. Hepatology.

[36] Jiang W., Wu N., Wang X., Chi Y., Zhang Y., Qiu X., Hu Y., Li J., Liu Y. (2015). Dysbiosis Gut Microbiota Associated with Inflammation and Impaired Mucosal Immune Function in Intestine of Humans with Non-Alcoholic Fatty Liver Disease. Sci. Rep..

[37] Michail S., Lin M., Frey M.R., Fanter R., Paliy O., Hilbush B., Reo N.V. (2015). Altered Gut Microbial Energy and Metabolism in Children with Non-Alcoholic Fatty Liver Disease. FEMS Microbiol. Ecol..

[38] Wang B., Jiang X., Cao M., Ge J., Bao Q., Tang L., Chen Y., Li L. (2016). Altered Fecal Microbiota Correlates with Liver Biochemistry in Nonobese Patients with Non-Alcoholic Fatty Liver Disease. Sci. Rep..

[39] Del Chierico F., Nobili V., Vernocchi P., Russo A., De Stefanis C., Gnani D., Furlanello C., Zandonà A., Paci P., Capuani G. (2017). Gut Microbiota Profiling of Pediatric Nonalcoholic Fatty Liver Disease and Obese Patients Unveiled by an Integrated Meta-Omics-Based Approach. Hepatology.

[40] Shen F., Zheng R.-D., Sun X.-Q., Ding W.-J., Wang X.-Y., Fan J.-G. (2017). Gut Microbiota Dysbiosis in Patients with Non-Alcoholic Fatty Liver Disease. Hepatobiliary Pancreat. Dis. Int..

[41] Da Silva H.E., Teterina A., Comelli E.M., Taibi A., Arendt B.M., Fischer S.E., Lou W., Allard J.P. (2018). Nonalcoholic Fatty Liver Disease Is Associated with Dysbiosis Independent of Body Mass Index and Insulin Resistance. Sci. Rep..

[42] Nistal E., Sáenz de Miera L.E., Ballesteros Pomar M., Sánchez-Campos S., García-Mediavilla M.V., Álvarez-Cuenllas B., Linares P., Olcoz J.L., Arias-Loste M.T., García-Lobo J.M. (2019). An Altered Fecal Microbiota Profile in Patients with Non-Alcoholic Fatty Liver Disease (NAFLD) Associated with Obesity. Rev. Espanola Enfermedades Dig. Organo Of. Soc. Espanola Patol. Dig..

[43] Yun Y., Kim H.-N., Lee E.-J., Ryu S., Chang Y., Shin H., Kim H.-L., Kim T.H., Yoo K., Kim H.Y. (2019). Fecal and Blood Microbiota Profiles and Presence of Nonalcoholic Fatty Liver Disease in Obese versus Lean Subjects. PLoS ONE.

[44] Tsai M.-C., Liu Y.-Y., Lin C.-C., Wang C.-C., Wu Y.-J., Yong C.-C., Chen K.-D., Chuah S.-K., Yao C.-C., Huang P.-Y. (2020). Gut Microbiota Dysbiosis in Patients with Biopsy-Proven Nonalcoholic Fatty Liver Disease: A Cross-Sectional Study in Taiwan. Nutrients.

[45] Al-Jashamy K., Murad A., Zeehaida M., Rohaini M., Hasnan J. (2010). Prevalence of Colorectal Cancer Associated with Streptococcus Bovis among Inflammatory Bowel and Chronic Gastrointestinal Tract Disease Patients. Asian Pac. J. Cancer Prev. APJCP.

[46] Gevers D., Kugathasan S., Denson L.A., Vázquez-Baeza Y., Van Treuren W., Ren B., Schwager E., Knights D., Song S.J., Yassour M. (2014). The Treatment-Naive Microbiome in New-Onset Crohn’s Disease. Cell Host Microbe.

[47] Forbes J.D., Chen C.-Y., Knox N.C., Marrie R.-A., El-Gabalawy H., de Kievit T., Alfa M., Bernstein C.N., Van Domselaar G. (2018). A Comparative Study of the Gut Microbiota in Immune-Mediated Inflammatory Diseases-Does a Common Dysbiosis Exist?. Microbiome.

[48] Chen Y., Yang F., Lu H., Wang B., Chen Y., Lei D., Wang Y., Zhu B., Li L. (2011). Characterization of Fecal Microbial Communities in Patients with Liver Cirrhosis. Hepatology.

[49] Ponziani F.R., Bhoori S., Castelli C., Putignani L., Rivoltini L., Del Chierico F., Sanguinetti M., Morelli D., Paroni Sterbini F., Petito V. (2019). Hepatocellular Carcinoma Is Associated with Gut Microbiota Profile and Inflammation in Nonalcoholic Fatty Liver Disease. Hepatology.

[50] Behari J., Graham L., Wang R., Schirda C., Borhani A.A., Methé B.A., Li K., Morris A., Luu H.N., Palmieri S. (2021). Dynamics of Hepatic Steatosis Resolution and Changes in Gut Microbiome with Weight Loss in Nonalcoholic Fatty Liver Disease. Obes. Sci. Pract..

[51] Santos-Marcos J.A., Haro C., Vega-Rojas A., Alcala-Diaz J.F., Molina-Abril H., Leon-Acuña A., Lopez-Moreno J., Landa B.B., Tena-Sempere M., Perez-Martinez P. (2019). Sex Differences in the Gut Microbiota as Potential Determinants of Gender Predisposition to Disease. Mol. Nutr. Food Res..

[52] Manor O., Dai C.L., Kornilov S.A., Smith B., Price N.D., Lovejoy J.C., Gibbons S.M., Magis A.T. (2020). Health and Disease Markers Correlate with Gut Microbiome Composition across Thousands of People. Nat. Commun..

[53] Goodrich J.K., Waters J.L., Poole A.C., Sutter J.L., Koren O., Blekhman R., Beaumont M., Van Treuren W., Knight R., Bell J.T. (2014). Human Genetics Shape the Gut Microbiome. Cell.

[54] Fu J., Bonder M.J., Cenit M.C., Tigchelaar E.F., Maatman A., Dekens J.A.M., Brandsma E., Marczynska J., Imhann F., Weersma R.K. (2015). The Gut Microbiome Contributes to a Substantial Proportion of the Variation in Blood Lipids. Circ. Res..

[55] Jackson M.A., Jackson M., Jeffery I.B., Beaumont M., Bell J.T., Clark A.G., Ley R.E., O’Toole P.W., Spector T.D., Steves C.J. (2016). Signatures of Early Frailty in the Gut Microbiota. Genome Med..

[56] Org E., Blum Y., Kasela S., Mehrabian M., Kuusisto J., Kangas A.J., Soininen P., Wang Z., Ala-Korpela M., Hazen S.L. (2017). Relationships between Gut Microbiota, Plasma Metabolites, and Metabolic Syndrome Traits in the METSIM Cohort. Genome Biol..

[57] Falony G., Joossens M., Vieira-Silva S., Wang J., Darzi Y., Faust K., Kurilshikov A., Bonder M.J., Valles-Colomer M., Vandeputte D. (2016). Population-Level Analysis of Gut Microbiome Variation. Science.

[58] Jackson M.A., Verdi S., Maxan M.-E., Shin C.M., Zierer J., Bowyer R.C.E., Martin T., Williams F.M.K., Menni C., Bell J.T. (2018). Gut Microbiota Associations with Common Diseases and Prescription Medications in a Population-Based Cohort. Nat. Commun..

[59] Vich Vila A., Collij V., Sanna S., Sinha T., Imhann F., Bourgonje A.R., Mujagic Z., Jonkers D.M.A.E., Masclee A.A.M., Fu J. (2020). Impact of Commonly Used Drugs on the Composition and Metabolic Function of the Gut Microbiota. Nat. Commun..

[60] Tap J., Cools-Portier S., Pavan S., Druesne A., Öhman L., Törnblom H., Simren M., Derrien M. (2019). Effects of the Long-Term Storage of Human Fecal Microbiota Samples Collected in RNAlater. Sci. Rep..

[61] Gavriliuc S., Stothart M.R., Henry A., Poissant J. (2021). Long-Term Storage of Feces at −80 °C versus −20 °C Is Negligible for 16S RRNA Amplicon Profiling of the Equine Bacterial Microbiome. PeerJ.

[62] Fianchi F., Liguori A., Gasbarrini A., Grieco A., Miele L. (2021). Nonalcoholic Fatty Liver Disease (NAFLD) as Model of Gut-Liver Axis Interaction: From Pathophysiology to Potential Target of Treatment for Personalized Therapy. Int. J. Mol. Sci..

