# Supplementary material for: Sex-Specific Associations between Gut Microbiome and Non-Alcoholic Fatty Liver Disease among Urban Chinese Adults
Source: Microorganisms. 2021 Oct 8;9(10):2118. doi: 10.3390/microorganisms9102118 (PMC8537656; doi:10.3390/microorganisms9102118)
Supplement: Supplementary file 1 [file microorganisms-09-02118-s001.zip › microorganisms-1409115-supplementary.pdf]

Online Supplementary Material

Shi J, Yang Y, Xu W, et al. **Sex-specific associations between gut microbiome and non-alcoholic fatty liver disease among urban Chinese adults**

Supplementary Figure S1. Flow chart of study participants inclusion and exclusion

Supplementary Table S1. Association of no-alcoholic fatty liver disease and gut microbial richness and α-diversity metrics.

Supplementary Table S2. Associations of long-term diet quality and intakes of major food groups with gut microbiome alpha diversity in the Shanghai Women's and Men's Health Studies

Supplementary Table S3. Associations of long-term diet quality and food intakes with common gut microbiome taxa in the Shanghai Women's and Men's Health Studies

Supplementary Table S4. Associations of long-term diet quality and food intakes with rare gut microbiome taxa in the Shanghai Women's and Men's Health Studies

Supplementary Figure S1. Flow chart of study participants inclusion and exclusion

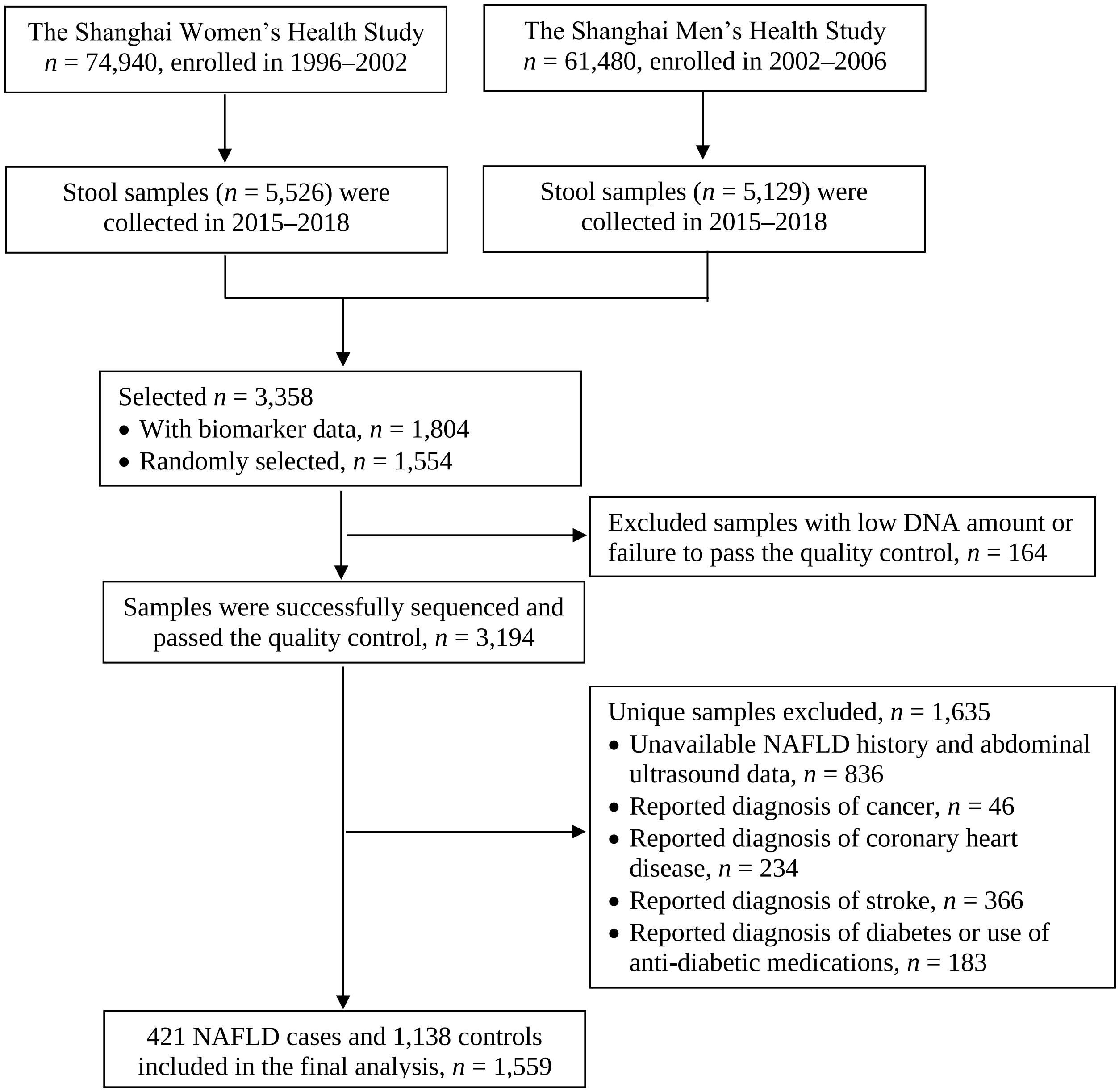

Supplementary Table S1. Association of no-alcoholic fatty liver disease and gut microbial richness and *α*-diversity metrics.

	NAFLD	Non-NAFLD	
	mean (SD)	mean (SD)	p [a]
SMHS (males)	n = 188	n = 571	
Microbial richness			
Number of detected OTUs	346.9 (102.1)	365.1 (90.0)	0.014
α-diversity			
Chao1	480.9 (137.6)	509.2 (121.6)	0.006
PD_whole_tree	24.0 (5.6)	25.1 (5.1)	0.020
Shannon index	4.58 (0.88)	4.76 (0.86)	0.015
SWHS (females)	n = 233	n = 567	
Microbial richness			
Number of detected OTUs	365.7 (92.6)	355.4 (93.3)	0.011
α-diversity			
Chao1	505.5 (121.8)	490.1 (125.7)	0.011
PD_whole_tree	25.1 (5.2)	24.5 (5.1)	0.006
Shannon index	4.77 (0.82)	4.73 (0.83)	0.030
Combined	n = 421	n = 1,138	
Microbial richness			
Number of detected OTUs	357.3 (97.3)	360.2 (91.8)	0.781
α-diversity			
Chao1	494.5 (129.5)	499.7 (124.0)	0.933
PD_whole_tree	24.6 (5.4)	24.8 (5.1)	0.634
Shannon index	4.69 (0.85)	4.74 (0.84)	0.949

[a] Comparison with non-alcoholic fatty liver disease controls using general linear regression, adjusted for age at stool sampling, the season of sample collection, gender (only for combined analysis), body mass index, waist-to-hip ratio, education, income, smoking status, alcohol drinking status, physical activity, total energy intake, fat intake, bowel movement frequency, history of hypertension, history of dyslipidemia, and sequencing batch.

NAFLD, non-alcoholic fatty liver disease; OTU, operational taxonomic unit; PD, phylogenetic diversity; SD, standard deviation; SMHS, Shanghai Men's Health Study; SWHS, Shanghai Women's Health Study.

Supplementary Table S2. Nominal association of non-alcoholic fatty liver disease and common bacterial taxa [a]

Taxon [b]	Non-NAFLD (n=1,138)	NAFLD (n=421)			
	Median RA (%)	Median RA (%)	Beta (se) [c]	*P* [c]	FDR [d]
p_*Firmicutes* ;c_*Bacilli* ;o_*Lactobacillales* ;f_*Streptococcaceae* ;g_*Streptococcus* ;s_*unclassified*	0.0628	0.0765	0.242 (0.106)	0.022	0.992
p_*Firmicutes* ;c_*Clostridia* ;o_*Clostridiale* s;f_*Lachnospiraceae* ;g_*Blautia*	1.2865	1.1491	-0.12 (0.06)	0.045	0.985
p_*Firmicutes* ;c_*Clostridia* ;o_*Clostridiale* s;f_*Lachnospiraceae* ;g_*Blautia* ;*s_unclassified*	1.1059	1.0224	-0.131 (0.063)	0.039	0.992

[a] The common taxa were defined as those with relative abundance ≥0.00588% and present in (carrier frequency) >50% of control participants.

[b] p_, c_, o_, f_, g_, and s_ indicate taxonomic levels of phylum, class, order, family, genus, and species, respectively.

[c] For each sample, centered log-ratio transformation was used to normalize taxa counts at each taxonomic level after adding a pseudo-count of 1. Beta, se and *p* values were calculated from general linear regression with NAFLD controls as reference, adjusted for age at stool sampling, sex, the season of sample collection, body mass index, waist-to-hip ratio, education, income, smoking status, alcohol drinking status, physical activity, total energy intake, fat intake, bowel movement frequency, history of hypertension, history of dyslipidemia, and sequencing batch.

[d] False discovery rate (FDR) <0.1 at each taxonomic level.

NAFLD, non-alcoholic fatty liver disease; RA, relative abundance; se, standard error.

Supplementary Table S3. Nominal association of non-alcoholic fatty liver disease and rare gut bacterial taxa [a]

Taxon [b]	non-NAFLD (n=1,138)	NAFLD (n=421)			
	**Carrier frequency (%)**	**Carrier frequency (%)**	**Beta (se)** [c]	***p*** [c]	**FDR** [d]
p_*Firmicutes* ;c_*Clostridia* ;o_*Clostridiales* ;f_*Lachnospiraceae* ;g_*Coprococcus* ;s_*eutactus*	37.9	40.9	0.268 (0.132)	0.042	0.992
p_*Firmicutes* ;c_*Clostridia* ;o_*Clostridiales* ;f_*Veillonellaceae* ;g_*Megasphaera*	22.4	30.2	0.492 (0.143)	5.7×10^{-4}	0.047
p_*Firmicutes* ;c_*Clostridia* ;o_*Clostridiales* ;f_*Veillonellaceae* ;g_*Megasphaera* ;s_*unclassified*	22.4	30.2	0.492 (0.143)	5.7×10^{-4}	0.071

[a] The rare taxa were defined as those with relative abundance ≥0.00588% and present in (carrier frequency) 10-50% of control participants.

[b] p_, c_, o_, f_, g_, and s_ indicate taxonomic levels of phylum, class, order, family, genus, and species, respectively.

[c] Logistic regression model for NAFLD association with rare taxa, adjusted for age at stool sampling, sex, the season of sample collection, body mass index, waist-to-hip ratio, education, income, smoking status, alcohol drinking status, physical activity, total energy intake, fat intake, bowel movement frequency, history of hypertension, history of dyslipidemia, sequencing batch, and sequencing depth.

[d] False discovery rate (FDR) <0.1 at each taxonomic level.

NAFLD, non-alcoholic fatty liver disease; RA, relative abundance; se, standard error.

Supplementary Table S4. Significant interactions between NAFLD and non-sex categorical variables with individual taxa associations among men or women

Taxon[a]	Non-NAFLD (n=571)	NAFLD (*n* = 188)			Non-NAFLD (*n* = 567)	NAFLD (*n* = 233)			***p*** for interaction [d]
	**Median RA (%)**	**Median RA (%)**	**Beta (se)** [b]	***p*** [b]	**Median RA (%)**	**Median RA (%)**	**Beta (se)** [b]	***p*** [b]	
	Age<65 years at in men (99 cases, 260 controls)				Age≥65 years in men (89 cases, 311 controls)				
p_*Actinobacteria* ;c_*Actinobacteria* ;o_*Bifidobacteriales* ;f_*Bifidobacteriaceae* ;g_*Bifidobacterium* ;s_*adolescentis*	0.0384	0.0893	1.149 (0.319)	3.6×10^{-4}	0.0475	0.0308	-0.175 (0.312)	0.575	3.9×10^{-4}
p_*Actinobacteria* ;c_*Actinobacteria* ;o_*Bifidobacteriales* ;f_*Bifidobacteriaceae* ;g_*Bifidobacterium* ;*Other*	0.0073	0.0196	0.955 (0.250)	1.6×10^{-4}	0.0080	0.0046	-0.190 (0.246)	0.441	4.9×10^{-4}
	BMI<24 in men (54 cases,309 controls)				BMI≥24 in men (134 cases, 262 controls)				
p_*Firmicutes* ;c_*Clostridia* ;o_*Clostridiales* ;f_*Lachnospiraceae* ;g_[*Ruminococcus*];s_*torques*	0.0159	0.0357	0.688 (0.287)	0.017	0.0185	0.0085	-0.360 (0.199)	0.071	7.9×10^{-4}
	Healthy diet score <24.5 in men (82 cases, 266 controls)				Healthy diet score ≥24.5 in men (106 cases, 305 controls)				
p_*Proteobacteria* ;c_*Betaproteobacteria*	2.8838	4.1102	0.402 (0.203)	0.048	2.5753	2.3399	-0.289 (0.192)	0.133	0.006
	WHR<0.8 in women (53 cases, 218 controls)				WHR≥0.8 in women (180 cases, 349 controls)				
p_*Bacteroidetes* ;c_*Bacteroidia* ;o_*Bacteroidales* ;f_*Bacteroidaceae* ;g_*Bacteroides* ;s_*plebeius*	0.0090	0.2929	1.266 (0.609)	0.039	0.0071	0.0085	-0.176 (0.343)	0.607	5.4×10^{-4}
	**Carrier frequency (%)**	**Carrier frequency (%)**	**Beta (se)** [c]	***p*** [c]	**Carrier frequency(%)**	**Carrier frequency(%)**	**Beta (se)** [c]	***p*** [c]	
	WHR<0.9 in men (61 cases, 283 controls)				WHR≥0.9 in men (127 cases, 288 controls)				
p_*Firmicutes* ;c_*Clostridia* ;o_*Clostridiales* ;f_*Lachnospiraceae* ;g_*Blautia* ;s_*producta*	21.6	41	1.047 (0.356)	0.003	27.8	18.9	-0.412 (0.287)	0.151	2.2×10^{-4}
	Without hypertension in men (154 cases, 492 controls)				With hypertension in men (34 cases, 79 controls)				
p_*Tenericutes*	30.3	16.9	-0.666 (0.256)	0.009	15.2	35.3	2.319 (0.852)	0.006	7.2×10^{-4}
p_*Tenericutes* ;c_*Mollicutes*	28.9	14.3	-0.854 (0.270)	0.002	12.7	29.4	1.404 (0.753)	0.062	0.001
p_*Tenericutes* ;c_*Mollicutes* ;o_*RF39*	28	14.3	-0.813 (0.270)	0.003	12.7	26.5	1.195 (0.737)	0.105	0.005

[a] p_, c_, o_, f_, g_, and s_ indicate taxonomic levels of phylum, class, order, family, genus, and species, respectively.

[b] For each sample, centered log-ratio transformation was used to normalize taxa counts at each taxonomic level after adding a pseudo-count of 1. Beta, se and *P* values of common taxa association were calculated from general linear regression with NAFLD controls as reference, adjusted for age at stool sampling, the season of sample collection, body mass index, waist-to-hip ratio, education, income, smoking status, alcohol drinking status, physical activity, total energy intake, fat intake, bowel movement frequency, history of hypertension, history of dyslipidemia, and sequencing batch.

[c] Logistic regression model for NAFLD association with rare taxa, adjusted for age at stool sampling, the season of sample collection, body mass index, waist-to-hip ratio, education, income, smoking status, alcohol drinking status, physical activity, total energy intake, fat intake, bowel movement frequency, history of hypertension, history of dyslipidemia, sequencing batch, and sequencing depth.

[d] False discovery rate (FDR) <0.1 at each taxonomic level.

NAFLD, non-alcoholic fatty liver disease; RA, relative abundance; se, standard error; SMHS, Shanghai Men's Health Study; SWHS, Shanghai Women's Health Study.
